# Aortic Valve Annular Features in Acromegaly—A Detailed Three-Dimensional Speckle-Tracking Echocardiographic Analysis from the MAGYAR-Path Study

**DOI:** 10.3390/jcm14227899

**Published:** 2025-11-07

**Authors:** Attila Nemes, Csaba Lengyel, Tamás Várkonyi, Zsuzsanna Valkusz, Krisztina Kupai

**Affiliations:** Department of Medicine, Albert Szent-Györgyi Medical School, University of Szeged, H-6725 Szeged, Hungary; lengyel.csaba@med.u-szeged.hu (C.L.); varkonyi.tamas@med.u-szeged.hu (T.V.); valkusz.zsuzsanna@med.u-szeged.hu (Z.V.); kupai.krisztina@med.u-szeged.hu (K.K.)

**Keywords:** aortic valve, annulus, three-dimensional, speckle-tracking, echocardiography, acromegaly

## Abstract

**Background**: Acromegaly, typically caused by growth hormone (GH)-secreting pituitary adenomas, leads to chronic GH and insulin-like growth factor-1 overproduction, resulting in significant cardiovascular complications, including left ventricular (LV) hypertrophy, myocardial fibrosis, diastolic/systolic LV dysfunction, and frequent valvular disease. Although aortic root dilation has been documented, the morphology and function of the aortic valve annulus (AVA) and its relationship with LV performance remain unexplored. **Methods**: The present study comprised a total of 31 patients with acromegaly, from which eight subjects were excluded due to inferior image quality. The remaining group of acromegalics consisted of 23 cases (mean age: 54.3 ± 14.5 years, 6 males). Their results were compared to 31 age- and gender-matched healthy subjects (mean age: 50.0 ± 7.4 years, 9 males). Cardiac assessment involved routine two-dimensional Doppler echocardiography and three-dimensional speckle-tracking echocardiography (3DSTE) to quantify basal regional and global longitudinal strains. Detailed planimetric measurements of AVA dimensions and its spatial displacement, called AVA plane systolic excursion (AAPSE), were also obtained. **Results**: Among 12 patients with inactive acromegaly, 7 patients (58%) showed larger end-systolic AVA area (AVA-A), while 5 patients (42%) had larger end-diastolic AVA-A. Among the 11 patients with active acromegaly, 3 patients (27%) had larger end-systolic AVA-A and 5 patients (45%) had larger end-diastolic AVA-A, while in 3 patients (27%) end-systolic and end-diastolic AVA-A proved to be equal. All end-systolic and end-diastolic AVA dimensions were tendentiously greater in acromegaly, with more pronounced values seen in the presence of an active disease. AAPSE was reduced both in all acromegaly patients and in those with active disease compared to controls. From LV strains, basal and global LV longitudinal strain (LS) and basal LV circumferential strain (CS) were similar when comparing acromegaly patients and those with active and inactive disorder to controls. However, basal and global LV-LS tended to be reduced, while basal LV-CS tended to be increased. Significantly increased global LV-CS were present in active acromegaly patients compared to inactive acromegaly patients and controls **Conclusions**: Significant aortic valve annular dilation is present in acromegaly, which is associated with its reduced spatial systolic displacement.

## 1. Introduction

Acromegaly, most commonly due to a pituitary growth hormone (GH)-secreting adenoma, is characterized by chronic excess of GH and insulin-like growth factor-1 (IGF-1). Beyond their metabolic effects, sustained GH/IGF-1 exposure drives a spectrum of cardiovascular abnormalities and increases morbidity and mortality [1]. Direct myocardial and vascular actions combine with indirect effects mediated by insulin resistance, diabetes mellitus, and dyslipidemia to shape the cardiovascular phenotype [2]. From a cardiac standpoint, left ventricular (LV) remodeling is typical. Biventricular hypertrophy with disproportionate septal thickening and interstitial fibrosis contributes to diastolic dysfunction, arrhythmogenesis, and—when advanced—heart failure and sudden cardiac death [3,4]. LV contractile impairment can occur despite increased wall thickness and may relate to altered calcium handling and changes in contractile protein expression [5]. Concomitant vascular involvement—endothelial dysfunction, increased arterial stiffness, and accelerated atherosclerosis—further amplifies afterload and risk, favoring hypertension in this population [5,6,7,8]. Contemporary imaging studies support that in acromegaly, LV diastolic dysfunction is mechanistically linked to augmented aortic stiffness, indicating an integrated myocardial–vascular remodeling process [8]. Valvular disease is also prevalent in acromegaly. Mitral and aortic leaflet thickening/degeneration and regurgitation have been reported, and valve injury appears to correlate with cumulative GH exposure and may be only partially reversible, in contrast to LV hypertrophy [8,9,10]. Modern speckle tracking echocardiography (STE) confirms high valvular burden—predominantly mitral, followed by aortic—and subclinical myocardial dysfunction [11]. With respect to the aortic root–aortic valve annulus (AVA)–LV outflow tract (LVOT) complex, a bidirectional relationship is biologically plausible: LV geometry and function shape aortic root/annular motion and dimensions, while annular/root mechanics feedback on LV workload and ventricular–arterial coupling [8,12]. In acromegaly, however, the AVA itself remains largely uncharacterized. To the best of our knowledge, no study has systematically evaluated AVA morphology, dimensions, or mechanics in acromegaly, nor its association with LV function. Limited, earlier echocardiography data reported that acromegaly is associated with significant enlargement of the aortic root diameters, particularly at the sinotubular junction and ascending aorta, beyond what had been previously described. Importantly, these changes suggest a structural remodeling of AVA–LVOT complex [8,12]. By linking aortic root dilation to altered LV workload and ventricular–arterial coupling, the paper introduces a mechanistic framework for myocardial–valvular–vascular interaction in acromegaly. Thus, it highlights that annular and aortic root abnormalities may represent an underrecognized, but integral component of acromegaly-related cardiomyopathy [12]. Case reports have described aortic root ectasia/dilation and severe aortic regurgitation in acromegaly, underscoring potential clinical significance, but they do not address annular function or LV interaction [13,14]. To the best of our knowledge, AVA biomechanics and LV–AVA coupling have not yet been investigated.

## 2. Patients and Methods

### 2.1. Patient Population

The present study comprised a total of 31 patients with acromegaly, from which 8 subjects were excluded due to inferior image quality. The remaining group of acromegalics consisted of 23 cases, all of them being in sinus rhythm (mean age: 54.3 ± 14.5 years, 6 males). Acromegaly was diagnosed according to guidelines: if serum human GH and/or IGF-1 concentration was above the diagnostic threshold. Inactive acromegaly was defined as patients previously diagnosed with acromegaly, but adequately treated (hypophysis transsphenoidal surgery). The biochemical control measuring serum IGF-1 3 months after the surgery indicates, whether acromegaly is active or inactive. The IGF-1 index was defined as the ratio of measured serum IGF-1 (ng/mL) and upper limit of normal (ULN) for IGF-1. ULN refers to the highest value of the laboratory reference range for a given analyte, adjusted for age and sex. For IGF-1, laboratories provide age- and sex-specific normative intervals based on their assay [1,2,8,15].

- If the IGF-1 index is ≤1.0, the value is within the normal range (biochemically controlled/inactive acromegaly).
- If the IGF-1 index is >1.0, the IGF-1 is elevated (active disease).

In our study, the IGF-1 index was 1.17 ± 1.05 in inactive acromegaly patients, while it was 1.71 ± 1.03 in active acromegaly patients.

All patients were treated and followed at the Division of Endocrinology, Department of Medicine, University of Szeged, which is responsible for the care of severe/rare endocrine disorders as a tertiary center in Southeast Hungary. Out of 31 acromegaly patients, 11 proved to have an active disease. Their findings were compared to those of age- and gender-matched healthy individuals. This population consisted of 31 healthy subjects (mean age: 50.0 ± 7.4 years, 15 males), who had no disease or pathology in their medical history; none of them were regular medication users, smokers, athletes, pregnant, or obese. The present investigation is a part of the **M**otion **A**nalysis of the heart and **G**reat vessels b**Y** three-dimension**A**l speckle-t**R**acking echocardiography in **Path**ological cases (**MAGYAR-Path**) **Study** (’magyar’ means ‘Hungarian’ in the Hungarian language). This present study was organized as a cooperation between the Endocrine Center and the 3D echocardiography working group at the Department of Medicine, University of Szeged, Hungary. The study was approved by the Institutional and Regional Human Biomedical Research Committee of the University of Szeged, Hungary (No. 71/2011 and updated versions). The study was conducted in accordance with the Declaration of Helsinki (as revised in 2013), and all acromegaly patients and controls gave informed consent.

### 2.2. Two-Dimensional Doppler Echocardiography

Routine 2D Doppler echocardiographic examination included assessment of left atrial and LV dimensions, volumes, and Simpson’s ejection fraction (EF), as well as Doppler-based analysis of LV diastolic function (determination of the E/A ratio) [16]. Color Doppler imaging was used for the assessment of valvular regurgitation and stenosis. For these purposes, the Toshiba Artida^TM^ echocardiography tool with a 1–5 MHz PST-30BT phased-array transducer (Toshiba Medical Systems, Tokyo, Japan) was used.

### 2.3. Three-Dimensional Speckle-Tracking Echocardiography

3DSTE studies were performed according to recent practices using the same cardiac ultrasound equipment with a 3D-capable PST-25SX matrix-array transducer (Toshiba Medical Systems, Tokyo, Japan) [17,18,19,20,21]. 3DSTE was conducted in two parts. As a first step, 3D echocardiographic data acquisitions were performed from the apical window following image optimization (gain, magnitude, etc.). For optimal images, 6 subvolumes during 6 heart cycles were acquired during breath-hold with constant RR intervals on the electrocardiogram. The subvolumes were stitched together by the software. As a second step, datasets were analyzed by the vendor-derived software called as 3D Wall Motion Tracking (version 2.7, Toshiba Medical Systems, Tokyo, Japan).

**Figure 1:**
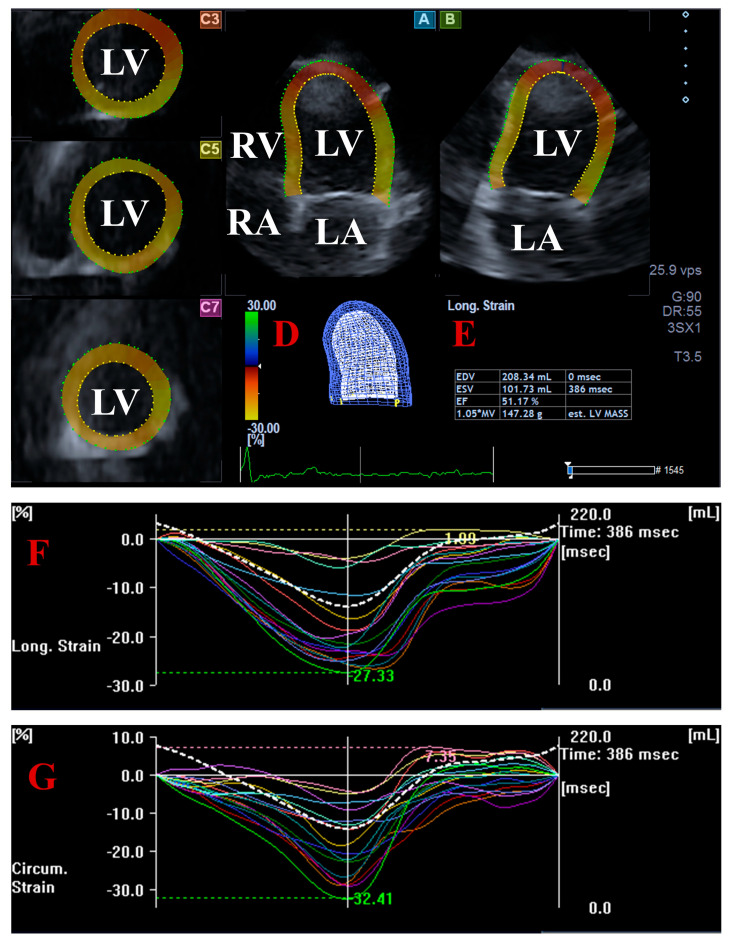
Measurement of left ventricular (LV) strains by three-dimensional (3D) speckle-tracking echocardiography: apical four-chamber (**A**) and two-chamber (**B**) long-axis views and short-axis views at apical (**C3**), midventricular (**C5**), and basal LV level (**C7**) are presented with a virtual 3D LV cast (**D**) and LV volumes and ejection fraction (**E**). Global (white line) and segmental (colored lines) time-longitudinal (**F**) and circumferential (**G**) time-LV strain curves are demonstrated together with a time–LV volume changes curve (dotted white line). Abbreviations: LV, left ventricle; RV, right ventricle; LA, left atrium; RA, right atrium; EF, ejection fraction; EDV, end-diastolic volume; ESV, end-systolic volume.

For LV strain analysis, together with apical 4-chamber (AP4CH) and 2-chamber (AP2CH) long-axis views, 3 cross-sectional views were automatically created. The septal and lateral edges of the LV-mitral annulus and the endocardial surface of the LV apex were determined by the observer, and following sequential analysis and automated contour detection, a virtual 3D model of the LV was created. The basal regional and global LV longitudinal strains (LSs), representing shortening/lengthening of the LV, and LV circumferential strains (LCs), representing narrowing/widening of the LV were measured [17,18,19,20,21] (Figure 1).

**Figure 2:**
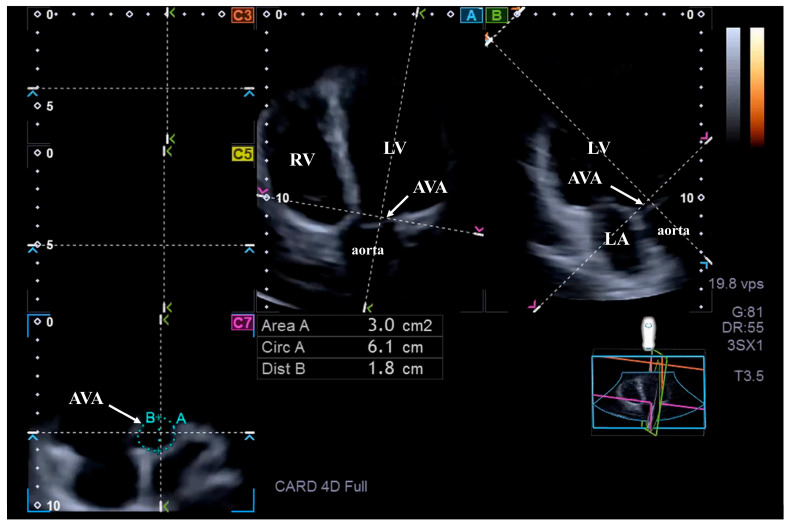
Measurement of the aortic valve annular dimensions by three-dimensional speckle-tracking echocardiography. Following optimization of the LV longitudinal planes on apical 4-chamber view (**A**) and 2-chamber view (**B**), and visualization of the aortic valve/aorta by tilting and optimizing the longitudinal planes in these long-axis views (on (**A**,**B**)). The planes were positioned to be parallel with the aortic root centerline. The C7 served as the cross-sectional view of the aortic valve annulus, which was perpendicular to the longitudinal plane. The white arrow represents the aortic valve annulus. Abbreviations: AVA = aortic valve annulus; Area = AVA area; Circ = AVA perimeter; Dist B = AVA diameter; LV, left ventricle; RV, right ventricle; LA, left atrium.

For AVA dimensions, optimal LV longitudinal planes were determined on AP4CH and AP2CH views. Following visualization of the aortic valve/aorta by tilting and optimizing the longitudinal planes in AP4CH and AP2CH views, the planes were positioned parallel to the aortic root centerline. The C7 cross-sectional view, to which the AVA was aligned, was perpendicular to the longitudinal plane. Special attention was given to ensure that C7 was truly perpendicular and that measurements were not taken at the level of the Valsalva or the LVOT. In our analysis, AVA was therefore measured at the basal insertion points of the aortic valve cusps, corresponding to the true anatomic AVA level. The following AVA characteristics were measured in end-diastole and end-systole: minimum and maximum AVA diameter (AVA-Dmin and AVA-Dmax, respectively), AVA area (AVA-A), and AVA perimeter (AVA-P), all measured during planimetry. Spatial displacement of the AVA plane during the cardiac cycle, represented by AVA plane systolic excursion (AAPSE), was also measured [22] (Figure 2).

### 2.4. Statistical Analysis

Data were presented as mean ± standard deviation (SD) or n (%), as appropriate. Homogeneity of variances was tested by Levene’s test. Analysis of datasets was carried out by independent sample *t*-test, analysis of variance (ANOVA), Fisher’s exact test, or Kruskal–Wallis H test, where appropriate. Bonferroni correction was applied for multiple comparisons. The Bland–Altman method was performed for intra- and interobserver agreement. To test the reproducibility of 3DSTE-derived AVA assessments, measurements were repeated twice by the same observer (intraobserver agreement) and by two observers (interobserver agreement) in 30 healthy individuals, together with their respective interclass correlation coefficients (ICCs). A *p*-value < 0.05 was considered statistically significant. Analyses were performed using SPSS version 29.0.0.0. (SPSS Inc., Chicago, IL, USA).

## 3. Results

### 3.1. Clinical Data

The most important demographic parameters and laboratory findings in acromegaly patients and controls are presented in Table 1. Parameters are shown separately for patients with active and inactive acromegaly as well.

### 3.2. Two-Dimensional Doppler Echocardiography

Greater LA and end-diastolic LV dimensions were observed in acromegaly, together with a thickened interventricular septum and LV posterior wall, compared with matched healthy controls. The presence of different grades of aortic, mitral, and tricuspid regurgitations is summarized in Table 2.

### 3.3. Three-Dimensional Speckle-Tracking Echocardiography

The mean frame rate for 3DSTE was 31 ± 3 fps. Out of 12 inactive acromegaly patients, 7 patients (58%) had larger end-systolic AVA-A, while 5 patients (42%) had larger end-diastolic AVA-A. Out of 11 active acromegaly patients, 3 patients (27%) had larger end-systolic AVA-A, and 5 patients (45%) had larger end-diastolic AVA-A, while in 3 patients (27%) end-systolic and end-diastolic AVA-A were equal. The ratio of subjects with greater end-diastolic AVA-A did not differ significantly between acromegaly patients and matched controls (10 out of 23 patients, 43%, vs. 12 out of 31 healthy controls, 39%). Similarly, subjects with equal-sized end-diastolic and end-systolic AVA-A were similar between the groups examined (3 out of 23, 13%, patients vs. 1 out of 31 healthy controls, 3%).

All end-systolic and end-diastolic AVA dimensions tended to be greater in acromegaly, with more pronounced values in the presence of active disease. A significant difference in maximum end-diastolic AVA diameter was detected between active acromegaly patients and controls. AAPSE was reduced in both acromegaly patients and those with active disease compared to controls. From LV strains, basal and global LV-LS and basal LV-CS were similar when comparing acromegaly patients and those with active and inactive disorder to controls. However, basal and global LV-LS tended to be reduced, while basal LV-CS tended to be increased. Significantly increased global LV-CS were present in active acromegaly patients compared to inactive acromegaly patients and controls (Table 3).

### 3.4. Reproducibility of 3DSTE-Derived AVA Assessments

Intraobserver and interobserver agreements of end-diastolic and end-systolic AVA diameters, areas, and perimeters are presented with their respective ICCs in Table 4.

## 4. Discussion

The insidious nature of acromegaly often leads to delayed diagnosis, which can exacerbate the severity of the cardiac manifestations, highlighting the importance of early detection to mitigate long-term cardiovascular damage and improve patient outcomes [22]. Specifically, the impact of acromegaly extends to both the cardiac chambers, influencing their dimensions and overall performance. LV end-diastolic dimensions are often increased in patients with acromegaly, reflecting dilation in response to chronic LV volume overload and impaired diastolic relaxation [8,23]. While LV hypertrophy is a common finding in acromegaly, eccentric hypertrophy, characterized by an increase in both LV mass and end-diastolic volume, is frequently observed in patients with depressed LV-EF. It develops in response to increased afterload in order to compensate for its wall stress and to maintain normal cardiac function [8,24]. The increase in LV end-diastolic dimension is not merely a consequence of volume overload, but also reflects intrinsic changes in the myocardial structure. Initially, LV hypertrophy represents an adaptive response to augmented cardiac performance; however, it carries the risk of heart failure or even sudden cardiac death, including cardiomyocyte hypertrophy, interstitial fibrosis, and impaired calcium handling [25]. The progression from LV hypertrophy to dysfunction involves complex mechanisms, with interstitial and replacement fibrosis playing a major role in the progressive decompensation of the hypertrophied LV. These alterations contribute to impaired diastolic relaxation, reduced systolic contractility, and ultimately, heart failure [26].

The pathophysiological background of valve disease in acromegaly is based on the unmediated effect of GH and IGF-I on connective tissue, manifesting as dysregulation in metalloproteinase expression, proteoglycan synthesis, as well as collagen and mucopolysaccharide deposition. Cardiac changes, while initially compensatory, can ultimately lead to significant valvular heart disease, thereby contributing to the increased cardiovascular morbidity and mortality observed in individuals with acromegaly. Valve abnormalities may be further aggravated by acromegaly-related aortic root dilatation, which has been reported in about 26% of the 42 patients in one study [27]. This condition was, in turn, positively correlated with LV mass. The findings of heart valve disease in acromegaly consist of aortic and mitral regurgitation. Colao et al. showed that 86% of the 42 studied active patients and 73% of the cured patients with acromegaly had valve disease, predominantly aortic valve regurgitation [9]. Van der Klaauw et al. conducted an observational study on 37 patients with active and controlled acromegaly and found that the prevalence of valve regurgitation increased during a mean follow-up of about 2 years [28]. Authors found a significant increase from baseline (56% to 88%) in active disease, and no change was found in controlled disease, further documenting the cumulative effect of GH overexposure on deterioration of valve function. The results also highlighted the irreversible yet stable character of this abnormality in inactive acromegaly. Additionally, investigators found, conversely to the study by Colao et al., no association between valve dysfunction and LV hypertrophy, suggesting that GH excess affects connective tissue rather than ventricular diameter as the underlying cause of this complication in the studied population [9].

The enormous development of cardiovascular imaging includes not only the emergence of new methods such as computer tomography and magnetic resonance imaging, but also the advancement of novel echocardiographic methods that are better suited for more accurate modeling of (patho)physiological processes. Although 3D echocardiography has been available for nearly 20 years, 3DSTE still holds considerable potential for uncovering new clinical data [17,18,19,20]. The suitability of 3D echocardiography for measuring AVA has been confirmed [29], and normal reference values for AVA measured with 3DSTE have been published with their age- and gender-dependency [21].

The findings of the present study have several implications. It was first demonstrated that AVA dimensions, their spatial displacement represented by AAPSE, and LV strains can be assessed simultaneously from an acquired 3D echocardiographic database during 3DSTE, even in the presence of a disease such as acromegaly. This enables deep pathophysiological analyses, such as the present study. Secondly, the ratio of acromegaly patients having greater end-diastolic than end-systolic AVA proved to be 43%, almost the same ratio as seen in healthy adults [21,22]. Its clinical consequence is not clear; further investigations are needed. Thirdly, it was confirmed that not only were the dimensions of the AVA dilated in the presence of acromegaly, but its spatial displacement, represented by AAPSE, was also reduced during the cardiac cycle. Although mostly tendentious differences could be detected, these abnormalities were more pronounced in the presence of an active disease. Finally, similar tendentious associations were found with contractility properties of the LV, including reduction in LV-LS representing shortening/lengthening and increase in LV-CS representing narrowing/widening of the global and basal regional LV in agreement with previous findings [7]. These abnormalities may raise the possibility of regional impairment of LV function, although a recently published study found no associations between the dimensions of AVA and AAPSE in healthy individuals [22]. It is rather possible that the contractile function in the evaluated patients with active acromegaly was rather changed (i.e., a change in the relative contribution of the LV-LS and LV-CS), but not globally reduced. This also corresponds to the fact that the LV-EF was not decreased, but rather slightly increased in the group of patients with active acromegaly. It is also worth considering the neighboring aortic root and its hemodynamic and elasticity characteristics [7]. Moreover, the difference between LV-EDV and LV-ESV was found to be high between all acromegaly patients and the control group (i.e., about 26% higher in patients with acromegaly), which suggests an important LV volume-overloading in the acromegaly group, which could only be partially explained by valvular incompetence. The questions raised above suggest the need for further investigations.

## 5. Limitation Section

- A limited number of patients with acromegaly were analyzed in this study. It should be noted that acromegaly is a rare disease, and the present study is a single-center study. However, in Hungary, with a population of less than 10 million, approximately 300 patients with acromegaly are followed, which means that about 10% of this population was examined in the present study.
- Disease activity was determined based on IGF-1 and IGF-1 index levels only, without considering GH suppression tests, which may have influenced the categorization of active and inactive acromegaly patients [15].
- Acromegaly-associated cardiovascular risk factors could partially explain the findings.
- One of the most important technical issues regarding 3DSTE is its low temporal and spatial resolution. Moreover, the footprint of the transducer for 3DSTE is greater than that of the transducer used for 2D echocardiography. In addition, the fact that more than one subvolume is necessary to be acquired for optimal images may result in stitching and motion artifacts and consequential deterioration of image quality [17,18,19,20].
- Although 3DSTE is capable of assessing all cardiac chambers and valvular annuli, the present analysis was restricted to AVA and LV parameters [17,18,19,20,21].
- Comparison of the determination of AVA dimensions by 2D Doppler echocardiography and 3DSTE was not the aim of this study.

## 6. Conclusions

This study is the first to demonstrate, using 3DSTE, that acromegaly is associated with significant dilation of the AVA, accompanied by its reduced systolic annular excursion (AAPSE). These alterations were more pronounced in patients with active disease compared to inactive acromegaly and healthy controls, indicating that chronic GH/IGF-1 excess promotes structural remodeling of the AVA–LV outflow tract complex and may impair valvulo-ventricular coupling. Our findings highlight the AVA as an underrecognized target of acromegaly-related cardiomyopathy and emphasize the need for further studies to clarify its clinical impact.

## Figures and Tables

**Table 1:** Clinical and demographic data of acromegaly patients and controls.

	Controls	All Acromegaly Patients	Inactive Acromegaly Patients	Active Acromegaly Patients
	(n = 31)	(n = 23)	(n = 12)	(n = 11)
**Clinical and demographic data**				
Age (years)	50.0 ± 7.5	54.3 ± 14.5	46.3 ± 15.0	63.2 ± 7.3 *^,†^
Male gender (%)	9 (29)	6 (26)	5 (42)	1 (9)
Hypertension (%)	0 (0)	13 (57) *	6 (50) *	7 (64) *
Diabetes mellitus (%)	0 (0)	4 (17)	2 (17)	2 (18)
Hypercholesterolemia (%)	0 (0)	11 (48) *	5 (42) *	6 (55) *
**Laboratory findings**				
Serum IGF-1 (ng/mL)	-	369.2 ± 324.5	345.4 ± 392.1	384.2 ± 234.5
Serum IGF-1 index	-	1.46 ± 1.07	1.17 ± 1.05	1.71 ± 1.03 ^†^

Abbreviations: IGF-1 = insulin-like growth factor-1. * *p* < 0.05 vs. controls; ^†^ *p* < 0.05 vs. inactive acromegaly patients.

**Table 2:** Echocardiographic parameters of acromegaly patients and controls.

	Controls	All Acromegaly Patients	Inactive Acromegaly Patients	Active Acromegaly Patients
	(n = 31)	(n = 23)	(n = 12)	(n = 11)
**LA and LV dimensions**				
LA diameter (mm)	38.7 ± 4.7	42.4 ± 5.8 *	41.2 ± 3.9 *	43.7 ± 7.1 *
LV-EDD (mm)	47.2 ± 3.5	51.0 ± 4.2	50.7 ± 4.2	51.4 ± 4.1
LV-EDV (mL)	104.1 ± 20.4	126.5 ± 23.4 *	126.2 ± 24.6	127.1 ± 22.1
LV-ESD (mm)	31.4 ± 3.1	31.2 ± 4.2	31.3 ± 3.4	31.1 ± 4.9
LV-ESV (mL)	35.3 ± 8.4	40.1 ± 12.8	40.5 ± 10.7	39.8 ± 14.6
IVS (mm)	9.4 ± 1.3	10.1 ± 1.4 *	9.7 ± 1.0	10.5 ± 1.6 *
LV-PW (mm)	9.7 ± 1.3	10.8 ± 1.6 *	10.7 ± 1.6 *	11.0 ± 1.6 *
LV-EF (%)	65.7 ± 3.3	68.2 ± 7.2	67.6 ± 5.1	68.8 ± 9.0
E (cm/s)	72.8 ± 15.6	69.0 ± 14.6	74.2 ± 16.6	62.8 ± 8.5
A (cm/s)	68.8 ± 17.8	81.6 ± 13.9 *	79.0 ± 15.3	84.8 ± 11.3 *
**Aortic valve regurgitation**				
grade 0 (%)	31 (100)	20 (87)	10 (83)	10 (91)
grade 1 (%)	0 (0)	2 (9)	2 (17)	0 (0)
grades 2–4 (%)	0 (0)	1 (4)	0 (0)	1 (9)
**Mitral regurgitation**				
grade 0 (%)	31 (100)	12 (52) *	8 (67) *	4 (36) *
grade 1 (%)	0 (0)	9 (39) *	4 (33) *	5 (45) *
grades 2–4 (%)	0 (0)	2 (9)	0 (0)	2 (18)
**Tricuspid regurgitation**				
grade 0 (%)	31 (100)	12 (52) *	9 (75) *	3 (27) *^,†^
grade 1 (%)	0 (0)	11 (48) *	3 (25) *	8 (73) *^,†^
grades 2–4 (%)	0 (0)	0 (0)	0 (0)	0 (0)

Abbreviations: LA = left atrial, LV = left ventricular, EDD = end-diastolic diameter, EDV = end-diastolic volume, ESD = end-systolic diameter, ESV = end-systolic volume, IVS = interventricular septum, PW = posterior wall, EF = ejection fraction, and E and A = early and late diastolic mitral inflow velocities. * *p* < 0.05 vs. controls; ^†^ *p* < 0.05 vs. inactive acromegaly patients.

**Table 3:** Aortic valve annulus (AVA) dimensions and left ventricular (LV) strain parameters of acromegaly patients and controls.

	Controls	All Acromegaly Patients	Inactive Acromegaly Patients	Active Acromegaly Patients
	(n = 31)	(n = 23)	(n = 12)	(n = 11)
**AVA dimensions**				
AVA-Dmax-D (cm)	2.05 ± 0.24	2.21 ± 0.38	2.14 ± 0.45	2.31 ± 0.22 *
AVA-Dmin-D (cm)	1.84 ± 0.26	1.97 ± 0.39	1.90 ± 0.44	2.07 ± 0.31
AVA-A-D (cm^2^)	3.22 ± 0.72	3.85 ± 1.36	3.88 ± 1.45	4.07 ± 1.19
AVA-P-D (cm)	6.41 ± 0.74	6.91 ± 1.22	6.73 ± 1.29	7.14 ± 1.06
AVA-Dmax-S (cm)	1.99 ± 0.28	2.17 ± 0.34	2.19 ± 0.39	2.14 ± 0.26
AVA-Dmin-S (cm)	1.88 ± 0.25	1.92 ± 0.29	1.92 ± 0.31	1.92 ± 0.26
AVA-A-S (cm^2^)	3.24 ± 0.81	3.60 ± 0.94	3.50 ± 1.04	3.73 ± 0.76
AVA-P-S (cm)	6.39 ± 0.87	6.77 ± 0.90	6.69 ± 0.98	6.87 ± 0.75
**AAPSE (cm)**	1.14 ± 0.22 ^†^	1.00 ± 0.28 *	1.03 ± 0.26	0.97 ± 0.29 *
**LV strains**				
basal LV-LS (%)	−21.2 ± 4.3	−19.1 ± 5.4	−20.3 ± 4.2	−17.9 ± 5.1
global LV-LS (%)	−16.4 ± 2.3	−15.6 ± 3.3	−16.9 ± 2.3	−15.8 ± 3.3
basal LV-CS (%)	−26.3 ± 6.0	−28.8 ± 5.1	−27.8 ± 4.6	−29.8 ± 5.4
global LV-CS (%)	−27.1 ± 6.0	−28.7 ± 4.5	−26.8 ± 4.3	−30.8 ± 3.5 *^,†^

Abbreviations: AVA = aortic valve annulus, Dmax = maximum AVA diameter, Dmin = minimum AVA diameter, A = AVA area, P = AVA perimeter, D = end-diastolic, S = end-systolic, AAPSE = AVA plane systolic excursion, LV = left ventricular, LS = longitudinal strain, and CS = circumferential strain. * *p* < 0.05 vs. controls; ^†^ *p* < 0.05 vs. inactive acromegaly patients.

**Table 4:** Intra- and interobserver variability for three-dimensional speckle-tracking echocardiography-derived assessment of aortic valve annular dimensions and aortic valve annular plane systolic excursion.

	Intraobserver Agreement		Interobserver Agreement	
	Mean ± 2SD Difference in Values Obtained by Two Measurements of the Same Observer	Correlation Coefficient Between Measurements of the Same Observer	Mean ± 2SD Difference in Values Obtained by Two Observers	Correlation Coefficient Between Independent Measurements of Two Observers
**AVA-Dmax-D (cm)**	−0.04 ± 0.19	0.87 (*p* < 0.01)	−0.07 ± 0.19	0.89 (*p* < 0.01)
**AVA-Dmin-D (cm)**	−0.02 ± 0.23	0.90 (*p* < 0.01)	−0.04 ± 0.25	0.92 (*p* < 0.01)
**AVA-A-D (cm^2^)**	−0.12 ± 0.58	0.93 (*p* < 0.01)	−0.12 ± 0.53	0.93 (*p* < 0.01)
**AVA-P-D (cm)**	−0.07 ± 0.64	0.91 (*p* < 0.01)	−0.12 ± 0.72	0.93 (*p* < 0.01)
**AVA-Dmax-S (cm)**	0.02 ± 0.30	0.91 (*p* < 0.01)	0.03 ± 0.34	0.93 (*p* < 0.01)
**AVA-Dmin-S (cm)**	0.08 ± 0.31	0.81 (*p* < 0.01)	0.04 ± 0.32	0.82 (*p* < 0.01)
**AVA-A-S (cm^2^)**	0.13 ± 0.71	0.92 (*p* < 0.01)	0.13 ± 0.73	0.93 (*p* < 0.01)
**AVA-P-S (cm)**	−0.02 ± 0.55	0.91 (*p* < 0.01)	0.02 ± 0.50	0.91 (*p* < 0.01)
**AAPSE (cm)**	−0.02 ± 0.22	0.92 (*p* < 0.01)	−0.02 ± 0.18	0.93 (*p* < 0.01)

Abbreviations: AVA = aortic valve annulus, Dmax = maximum AVA diameter, Dmin = minimum AVA diameter, A = AVA area, P = AVA perimeter, D = end-diastolic, S = end-systolic, AAPSE = AVA plane systolic excursion.

## Data Availability

The data presented in this study are available on request from the corresponding author.
